# Micrometer Scale Resolution Limit of a Fiber-Coupled Electro-Optic Probe

**DOI:** 10.3390/s19132874

**Published:** 2019-06-28

**Authors:** Young-Pyo Hong, Kyung-Min Lee, Sung-Yeol Kim, Meehyun Lim, Taekjin Kim, Hyung-Jung Yong, Dong-Joon Lee

**Affiliations:** 1 Division of Physical Metrology, Korea Research Institute of Standards and Science, Yuseong-gu, Daejeon 34113, Korea; 2 Manufacturing Technology Center, Samsung Electronics Co., Ltd., Hwaseong-si 18448, Korea

**Keywords:** electro-optic probe, fiber-optic sensor, image resolution, microwave measurement, microwave photonics

## Abstract

We present the practical resolution limit of a fine electrical structure based on a fiber-coupled electro-optic probing system. The spatial resolution limit was experimentally evaluated on the sub-millimeter to micrometer scale of planar electrical transmission lines. The electrical lines were fabricated to have various potential differences depending on the dimensions and geometry. The electric field between the lines was measured through an electro-optic probe, which was miniaturized up to the optical bare fiber scale so as to investigate the spatial limit of electrical signals with minimal invasiveness. The experimental results show that the technical resolution limitation of a fiber-coupled probe can reasonably approach a fraction of the mode field diameter (~10 μm) of the fiber in use.

## 1. Introduction

Electronic components have been miniaturized to interconnection scales that often reach sub-micrometer dimensions. To diagnose the health of fabricated devices/components, numerous measurement techniques have been developed. The wafer prober is one of the most widely used test solutions to find functional defects in integrated circuits on wafers. This solution requires a set of microscopic contacts or probes called a probe card that is held in place while the wafer on a chuck is moved beneath the probes to make an electrical contact. By applying such a special test pattern of movement, a customized and efficient test environment for industry can be realized. Because this solution relies on mechanical contacts on designated test pads, fast and reliable failure tests between test points are feasible. Hence, the probe card has become a mature and suitable technique for the manufacturing of integrated circuits.

In the early stage of integrated circuit design, another issue in addition to the connectivity test was the electromagnetic performance between the pads. Conventional electrical measurement techniques, including the use of a probe card, do not reliably provide this information, as the probe body is made of a metallic material, and its scale far exceeds the circuitry dimension of integrated electronic devices [1]. An alternative and well-established measurement solution is the electro-optic (EO) sensing technique associated with dielectric EO media [2]. The EO technique enables extremely fine resolutions associated with ultrathin EO media and precision optics [3]. A resolution of 2.7 μm incorporated with a 1.5 μm EO wafer in a free-space configuration has been reported [4]. 

In many practical applications, EO probes are usually coupled with optical fiber to realize the entire probe with an all-dielectric structure and enhance the convenience of use [5]. The EO material tips can be minutely diced to the scale of the bare optical fiber end [6,7]. Test methods with such EO probes on the optical fiber scale are believed to provide a minimally invasive solution as well as the ultimate spatial resolution [8]. In this paper, we experimentally explore the technical resolution limit of a fiber-coupled EO probe.

## 2. Experiment with Various Planar Transmission Lines

**Figure 1:**
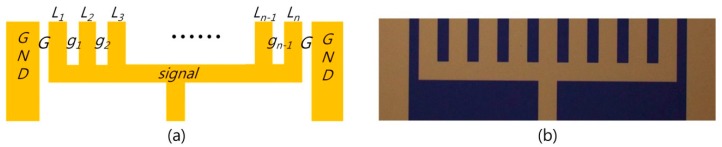
Multiline coplanar waveguide structure: (**a**) basic structure, and (**b**) fabricated example (*n* = 9, *L* = 100 μm, *g* = 60 μm, and *G* = 52 μm).

To explore the measurable spatial resolution limit of the minimally invasive EO probing system, we fabricated various transmission lines on a Si wafer. Figure 1 outlines the general test lines of interest in this case. It shows a basically coplanar waveguide (CPW) structure where the signal is guided between a pair of symmetric ground planes on a planar substrate. We changed the number of lines (*n*), line width (*L*), line-gap width (*g*), and the ground-gap width (*G*) and added intentional defects to certain lines. 

### 2.1. Single Coplanar Transmission Line

**Figure 2:**
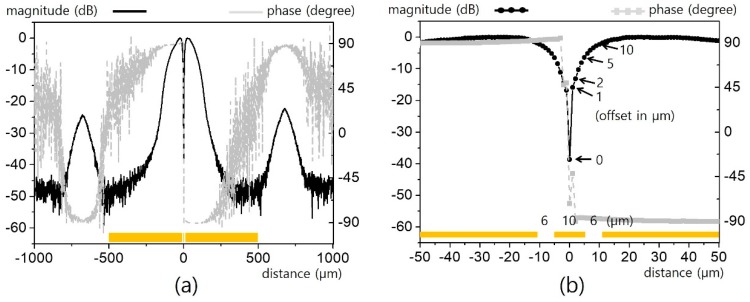
Horizontal electric field distribution over a coplanar waveguide (CPW) (*n* = 1, *L* = 10 μm, and *G* = 6 μm): (**a**) macroscopic view, and (**b**) microscopic view.

First, we considered the simplest CPW line (i.e., *n* = 1). The CPW was fed using a GSG-type on-wafer probe with a pitch of 150 μm. Power of 16 dBm at 100 kHz (for good sensitivity with lock-in detection) was launched onto the CPW at the designated port for on-wafer feeding. The dimensions and sensing method of our micro-EO probe, as well as the results of an invasive analysis of the fine structure, are reported in the literature [8]. The probe used here was made of an *x*-cut LiTaO_3_ wafer 50 μm thick, which was positioned as close as ~10 μm over the CPW for translation with a programmable servo-motion stage. The narrowest signal line we tested had dimensions of *L* = 10 μm and *G* = 6 μm, with these results presented in Figure 2. The EO probe translated in 20,000 steps with a precise resolution of 1 μm. The magnitude was normalized, and its signal level was at least 45 dB above the noise level. The magnitude-phase plot shows a typical electric field pattern for a CPW, where intense electric fields are symmetrically formed between signal and ground lines with opposite phases. In fact, the minimally detectable field strength of such a minute EO probe is typically at the level of a few V/m [7]. Despite such low sensitivity, the field strength associated with such a fine CPW is extremely intense owing to the narrow signal-to-ground gaps. 

We assumed that the effective spatial resolution would be comparable to the mode field diameter (MFD) of the optical fiber used because the majority of the beam was confined to that area and beam divergence was negligible for the 50-μm-thick wafer and the numerical aperture of the fiber. However, the experiment conducted here shows that our micro-EO probe can measure structures finer than the MFD. For instance, Figure 2b exhibits the apparent horizontal field pattern for a CPW across 6 μm gaps. Between the gaps, an extremely abrupt field flip-over arises at the center of the signal line. Even probe translation of 1 μm causes magnitude changes exceeding 20 dB.

**Figure 3:**
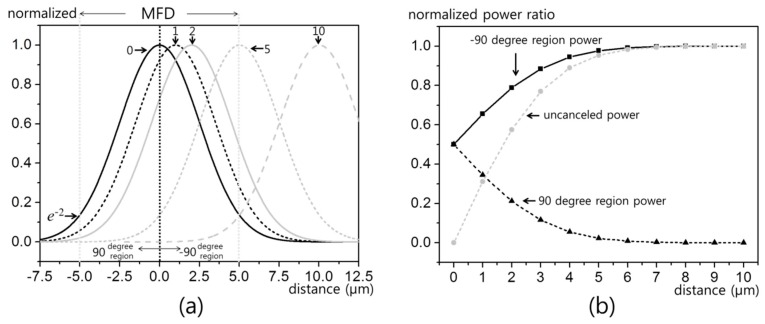
Sub-MFD (mode field diameter) resolution analysis in Figure 2: (**a**) normalized Gaussian beam profile for μm scale offset, and (**b**) beam portion versus phase.

Such fine sub-MFD resolutions can be quantitatively explained by beam profile analysis as shown in Figure 3. Figure 3a is the normalized Gaussian beam profile for 10 μm MFD. The shifted profiles are shown for the five offsets in Figure 2b. For 0 μm offset, the left and right half sides of the beam are precisely balanced. As each side has equal signal contribution with opposite phases, the EO signal becomes ideally canceled out. However, such a delicate symmetry breaks even for a minute offset. For instance, 1 μm offset causes 31% of uncanceled 90-degree component, which is enough to cause significant signal difference. The evolution of beam portion versus μm offset is presented in Figure 3b. It should be noted that just an offset of a half MFD (5 μm) causes 95% of uncanceled portion. This explains the nature of sub-MFD spatial resolution in a fiber-coupled EO probe. 

### 2.2. Triple Equipotential Coplanar Transmission Line

In many practical applications, multisignal lines are inevitable for system integration. For a larger scale of integration, the electrical lines must be narrower and closer. Unlike the single CPW line, the signal lines in this case are formed in a parallel bus to ensure multiple access capability and for faster transmission. To investigate the resolution of the multiline structure, we fabricated triple lines (*n* = 3 for Figure 1) with various scales (*L* = 100/50/30/10 μm). Each line is equipotential at 100 kHz as each was split from the same feed line. 

**Figure 4:**
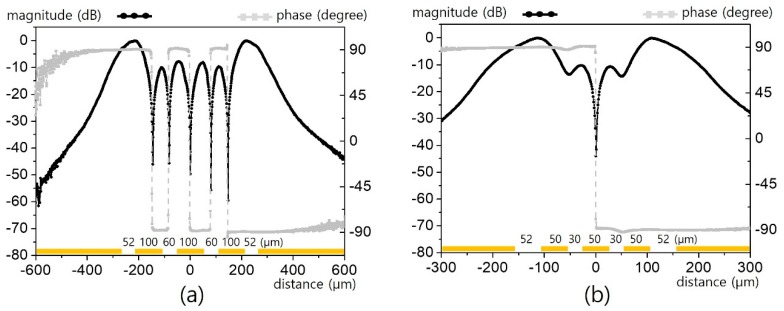
Horizontal electric field distribution over wide triple (*n* = 3) CPW lines: (**a**) for *L/g/G* = 100/60/52 μm, and (**b**) for *L/g/G* = 50/30/52 μm.

For relatively wide signal and gap lines (100 and 60 μm each as in Figure 4a), the apparent field pattern with a substantial contrast was measured. Given that *L*_2_ is sandwiched by symmetric equipotential lines (*L*_1_/*L*_3_), the electric field is weak due to the minute potential difference, while *L*_1_ and *L*_3_ show greater field patterns near the ground edges. Despite the fact that the field strength is not uniform, the three signal lines each cause an apparent dual peak. The six peaks with different magnitudes and phases shown in Figure 4a indicate number of signal lines and the relative distances from the ground. However, such clear contrast information becomes ambiguous as the line and gap scale is reduced to half, as shown in Figure 4b. The four apparent peaks above the three lines degraded to two blurred peaks. In addition, quite mild phase ripples over the two gaps among the signal lines serve as a clue with regard to the triple line characteristics.

**Figure 5:**
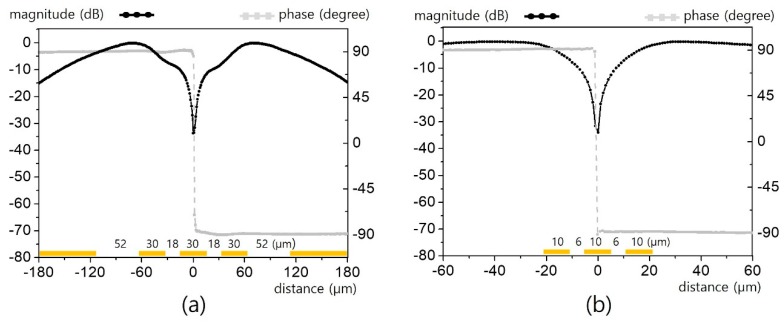
Horizontal electric field distribution over narrow triple (*n* = 3) CPW lines: (**a**) for *L/g/G* = 30/18/52 μm, and (**b**) for *L/g/G* = 10/6/52 μm.

The results of a further experiment with lines on a reduced scale, as shown in Figure 5a, can arguably infer evidence of three lines marginally. A main deep area always remains in the center of the lines, but other adjacent deep areas erode out for finer scales. For instance, the triple line with the same line-gap scale in Figure 2 is seen as a single line, as measured in Figure 5b. Such resolution degradation is not primarily because of the multiline feature as opposed to the challenges of EO probing. This will be discussed at the end of the paper.

### 2.3. Nine Equipotential Coplanar Transmission Lines with Artifacts

**Figure 6:**
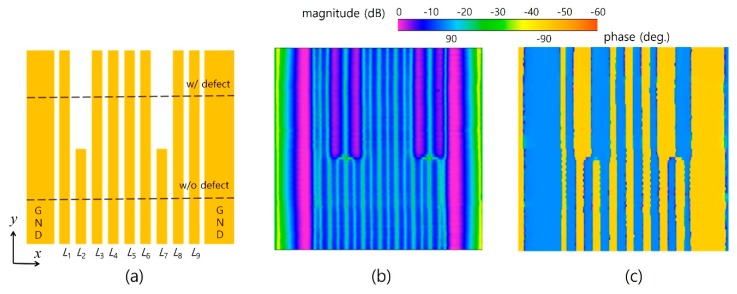
Horizontal electric field distribution over nine (*n* = 9) CPW lines (*L/g/G* = 100/60/52 μm) with artifacts: (**a**) layout, (**b**) magnitude image, and (**c**) phase image.

For signal transmission, multibus lines with uniform line widths and symmetry are often used. In a further investigation for more practical cases, we tested nine equipotential lines (*n* = 9) with intentional artifacts, as shown in Figure 6a. For visual insight into the line connectivity or device operation characteristics, the 2D field imaging technique is used [6]. For the line and gap widths of 100 and 60 μm and with the geometry shown in Figure 6a, the electric field distribution of the lines is shown in Figure 6b,c. The probe was translated over the lines on the planar *x*–*y* surface with transverse and longitudinal steps of 2 and 20 μm. The intentional artifacts in *L*_2_ and *L_7_* clearly observed are both the magnitude and phase distributions. 

**Figure 7:**
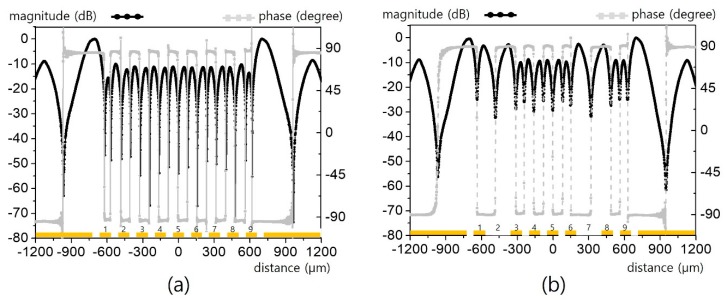
Horizontal electric field distribution over nine (*n* = 9, *L/g/G* = 100/60/52 μm) CPW lines in Figure 6: (**a**) for lines without defects, and (**b**) for line with defects.

**Figure 8:**
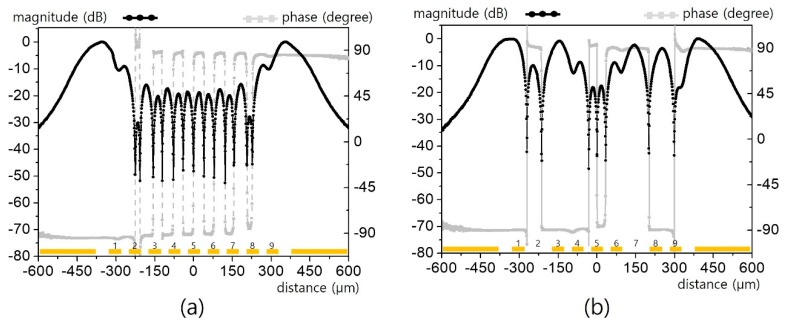
Horizontal electric field distribution over nine (*n* = 9, *L/g/G* = 50/30/52 μm) CPW lines in Figure 6: (**a**) for lines without defects, and (**b**) for lines with defects.

For a closer inspection, the 1D transverse scan for the respective lines in Figure 6a for *L* = 100 μm and *g* = 60 μm is presented in Figure 7. As in Figure 4, 9 lines with a width of 100 μm were all clearly distinguished, as were two artifacts. However, for the lines with a width of 50 μm in Figure 8, the field pattern becomes obscure from the edge sides (*L_1_*, *L_9_*). Moreover, the field patterns with artifacts are quite different after only scaling down the line width by half. Based on the magnitude and phase distribution in Figure 7b, the number of lines and their deployment can be clearly specified, whereas Figure 8b is obscure. In fact, Figure 8b is a sort of marginally feasible case to infer line deployment. Finer or more complex lines will increase the ambiguity of a multisignal line analysis.

### 2.4. Arbitrary Equipotential Coplanar Transmission Line

**Figure 9:**
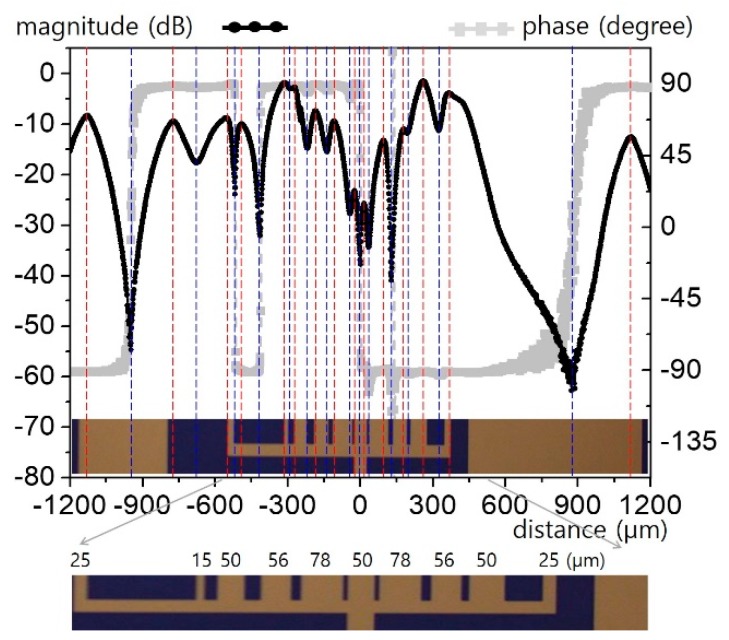
Horizontal electric field distribution over an arbitrary CPW (*n* = 10; detailed dimensions are given at the bottom).

For more practical and sophisticated circuitry, multiple lines with an arbitrary geometry must be considered. Figure 9 is an example of such lines. The lines are split from the single 50 μm feed line in the center. Phase information is mostly washed out owing to interferometric cancellations among the adjacent lines. However, the complicated ripples provide valuable information with which to infer the line geometry. The position and relative distance between the peak (red) and valley (blue) are indicated with colored lines in Figure 9, providing primary information about the line locations, widths, and separation distances. The magnitude and contrast of the ripples also correspondingly indicate the field strength and degree of interference among the lines.

### 2.5. Interdigital Line with Alternate Potential

**Figure 10:**
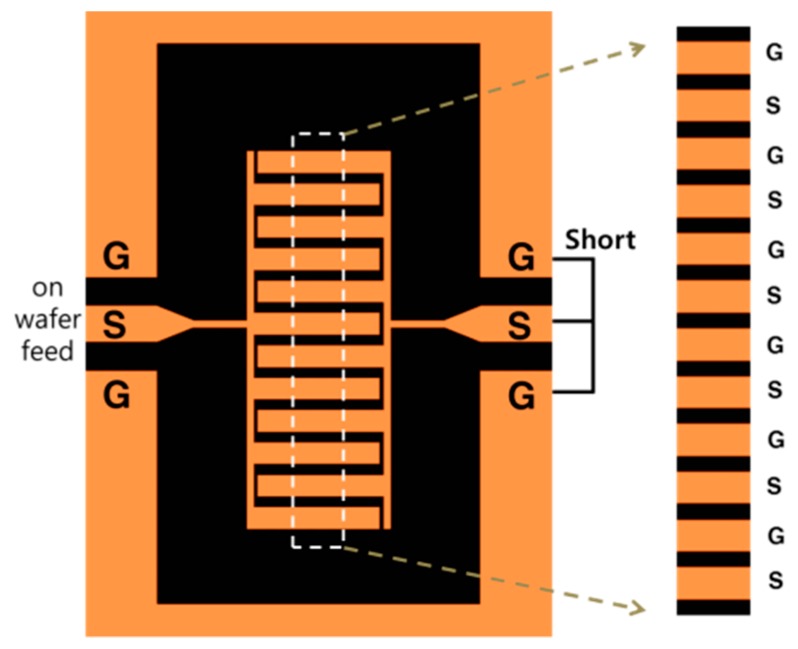
Geometry of the interdigital capacitor (detailed information is in the literature (ref. [9])).

Unlike equipotential lines, the electric field can be condensed by alternating multiple signal–ground lines. One widely used scheme uses interdigital lines. We employed the basic interdigital capacitor shown in Figure 10 [9]. The signal and ground line widths are identical, but the gap between them is designed to be half this distance. 

**Figure 11:**
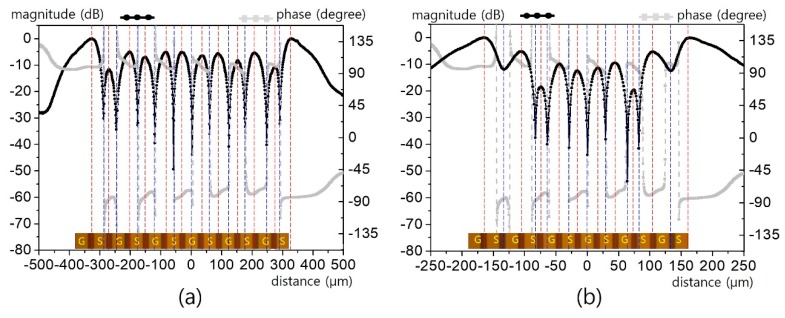
Horizontal electric field distribution for the interdigital lines in Figure 10: (**a**) for a 40 μm line width, (**b**) for a 20 μm line width.

**Figure 12:**
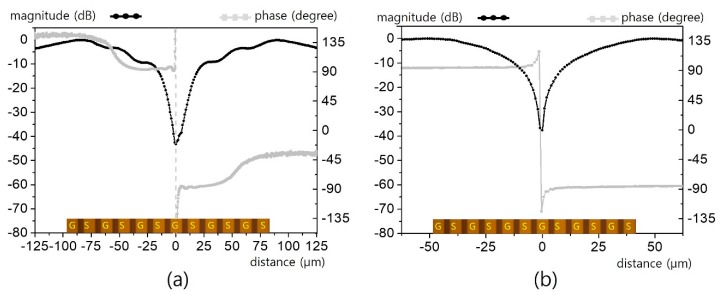
Horizontal electric field distribution for the interdigital lines in Figure 10: (**a**) for a 10 μm line width, (**b**) for a 5 μm line width.

The field patterns of three interdigital capacitors for respective line widths of 40/20/10/5 μm are presented in Figure 11 and Figure 12. For a 40 μm line (Figure 11a), clear magnitude and phase contrast can be observed sufficiently to specify all of the lines. Although the apparent contrast is degraded for the 20 μm line (Figure 11b), it is still decent enough to identify the interdigitated structure. However, such contrast is eroded overall for the 10 μm line (Figure 12a) and completely washed out for the 5 μm line (Figure 12b).

**Figure 13:**
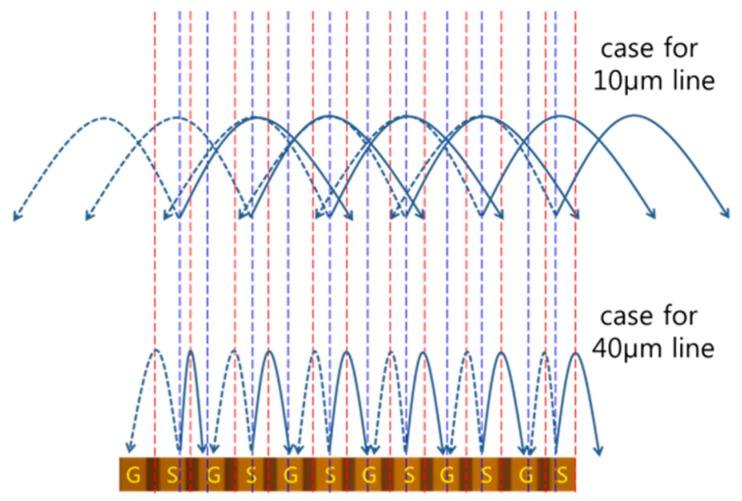
Qualitative electric field flux for the results shown in Figure 11 and Figure 12 (dashed line is to distinguish the phase).

The experimental data provide a practically technical limit of the spatial resolutions by EO probing for multiple signal lines with various potential differences. The resolution tends to degrade rapidly as line integrity increases. However, such spatial resolutions do not originate from measurement limits based on the proposed EO probing method. When the electrical lines and gaps are wide enough to be considered as distinct, the majority of the electric field flux from the signal sinks into the most adjacent ground lines. Therefore, the chance of interference is quite low, as illustrated in the bottom part of Figure 13. 

As opposed to this, the flux change for a four-fold narrower case is shown on the top side of Figure 13. The electric fields are mostly canceled out at the center of the line. Such a complex interference effect is mitigated toward both edge sides. Extremely narrow lines appear as virtually a single line, and the measured field resembles a simple CPW field, as shown in Figure 2. Therefore, the resolution is not limited primarily by the EO probing capability. For a simple CPW case in which a sharp field pattern exists without interference, the actual resolution is even finer than the mode field diameter of the optical fiber in use.

## 3. Conclusions

The feasible resolution limit of fiber-coupled electro-optic probing is experimentally investigated through various planar electrical lines. The probe itself can have technically 1 μm of spatial resolution. Such a limit heavily depends on the electrode patterns and pitch dimensions rather than probes. The results presented here can serve as a useful guideline when the spatial resolution is a great concern in measurements of miniaturized components.
